# Nanoporous gold nanoleaf as tunable metamaterial

**DOI:** 10.1038/s41598-021-81128-4

**Published:** 2021-01-19

**Authors:** Sangeeta Rout, Zhen Qi, Monika M. Biener, Devon Courtwright, Jakeem C. Adrien, Ezekiel Mills, Mohammad Shahabuddin, Natalia Noginova, Mikhail A. Noginov

**Affiliations:** 1 Center for Materials Research, Norfolk State University, Norfolk, VA 23504 USA; 2 Lawrence Livermore National Laboratory, Livermore, CA 94550 USA; 3 Virginia State University, Petersburg, VA 23806 USA

**Keywords:** Materials science, Optics and photonics, Physics

## Abstract

We have studied optical properties of single-layer and multi-fold nanoporous gold leaf (NPGL) metamaterials and observed highly unusual transmission spectra composed of two well-resolved peaks. We explain this phenomenon in terms of a surface plasmon absorption band positioned on the top of a broader transmission band, the latter being characteristic of both homogeneous “solid” and inhomogeneous “diluted” Au films. The transmission spectra of NPGL metamaterials were shown to be controlled by external dielectric environments, e.g. water and applied voltage in an electrochemical cell. This paves the road to numerous functionalities of the studied tunable and active metamaterials, including control of spontaneous emission, energy transfer and many others.

## Introduction

Complex metal-dielectric environments, including metamaterials^1–3^, plasmonic structures^4–6^, waveguides^7–9^ and cavities^10–12^, have been demonstrated to control scores of physical phenomena including spontaneous^13–22^ and stimulated^23–27^ emission, Förster energy transfer^11,28,29^, van der Waals interactions^30,31^, and chemical reactions^32–37^. Of particular interest are tunable and active metamaterials allowing one to manipulate light and other physical processes in real time^38–40^.

Transmission is one of the most important characteristics of optical materials. An extraordinary large transmission^41–43^, facilitated by plasmons, has been observed in arrays of subwavelength holes in metallic films. Other nanostructured composite metal/dielectric materials with remarkable properties include, among others, arrays of metallic nanowires grown in channels of porous alumina membranes^44,45^, nanoporous Au foams^46,47^, gyroidal structures^48^, etc.

At this time, we report studies and control of optical properties of nanoporous gold leafs (NPGLs)—unique inch-size single-layer and multi-fold (meta)materials, whose thickness can be varied between ~ 0.1 and ~ 1 µm. Composite materials with nominally similar composition and morphology have been studied in the literature^46,49–53^. In particular, it has been shown that their optical responses can be tuned by external stimuli and environments^49,50^, enabling multiple applications of active and tunable metamaterials. At the same time, the optical properties of NPGLs reported by different research groups^49,50,52,54^ were quite different from each other and the intriguing spectra of these porous materials, featuring e.g. the double-headed transmission band, were never clearly understood. This motivated our studies of NPGLs reported below.

## Experimental samples

The NPGL samples have been prepared from one layer of 12 K “white gold” leaf (50 wt.% Au and 50 wt.% Ag alloy, from Gold Leaf Company) through dealloying in 10 ml of concentrated HNO_3_ (68%, ACS, VWR) solution at room temperature for one hour. After complete removal of Ag, the volume filling factor of Au was 35 vol.%. The area of the nanoporous Au leafs was of the order of several centimeters squared.

**Figure 1:**
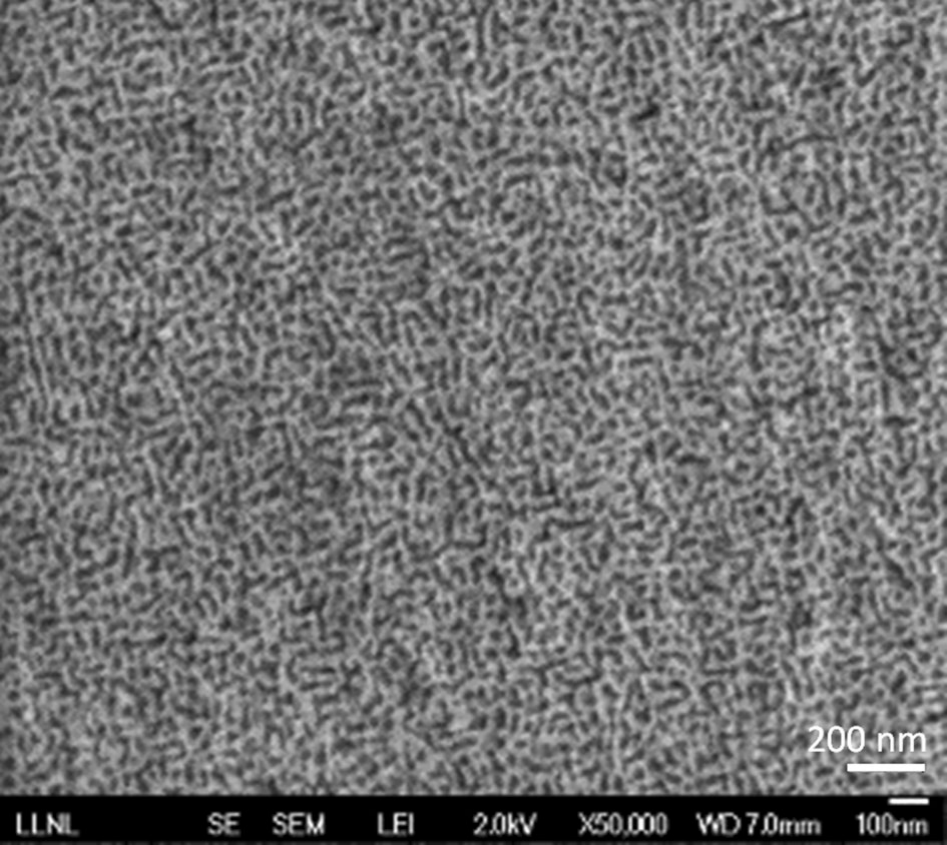
Scanning electron microscope (SEM) image of the single layer nanoporous gold leaf sample.

The scanning electron microscope (SEM) image of the single-layer Au nanoleaf sample is depicted in Fig. 1. The dealloying parameters for all samples in this work were nominally the same, resulting in ligaments’ diameters in different samples studied (determined using the Image J software) to range from 20 ± 3 nm to 27 ± 3 nm. The NPG leafs were rinsed thoroughly in deionized (DI) water for at least three times after the dealloying process before being transferred onto microscope glass slides. Different thicknesses of the fabricated samples, ranging from 84 to 943 nm, were achieved by using a single NPGL layer or folding the gold leaf once (two layers), twice (four layers), and three times (eight layers). The film’s thickness was measured using the stylus profilometer (DekTak XT form Bruker). The fabricated samples were dried in air before taking any measurements.

## Results and discussion

### Transmission and reflection

**Figure 2:**
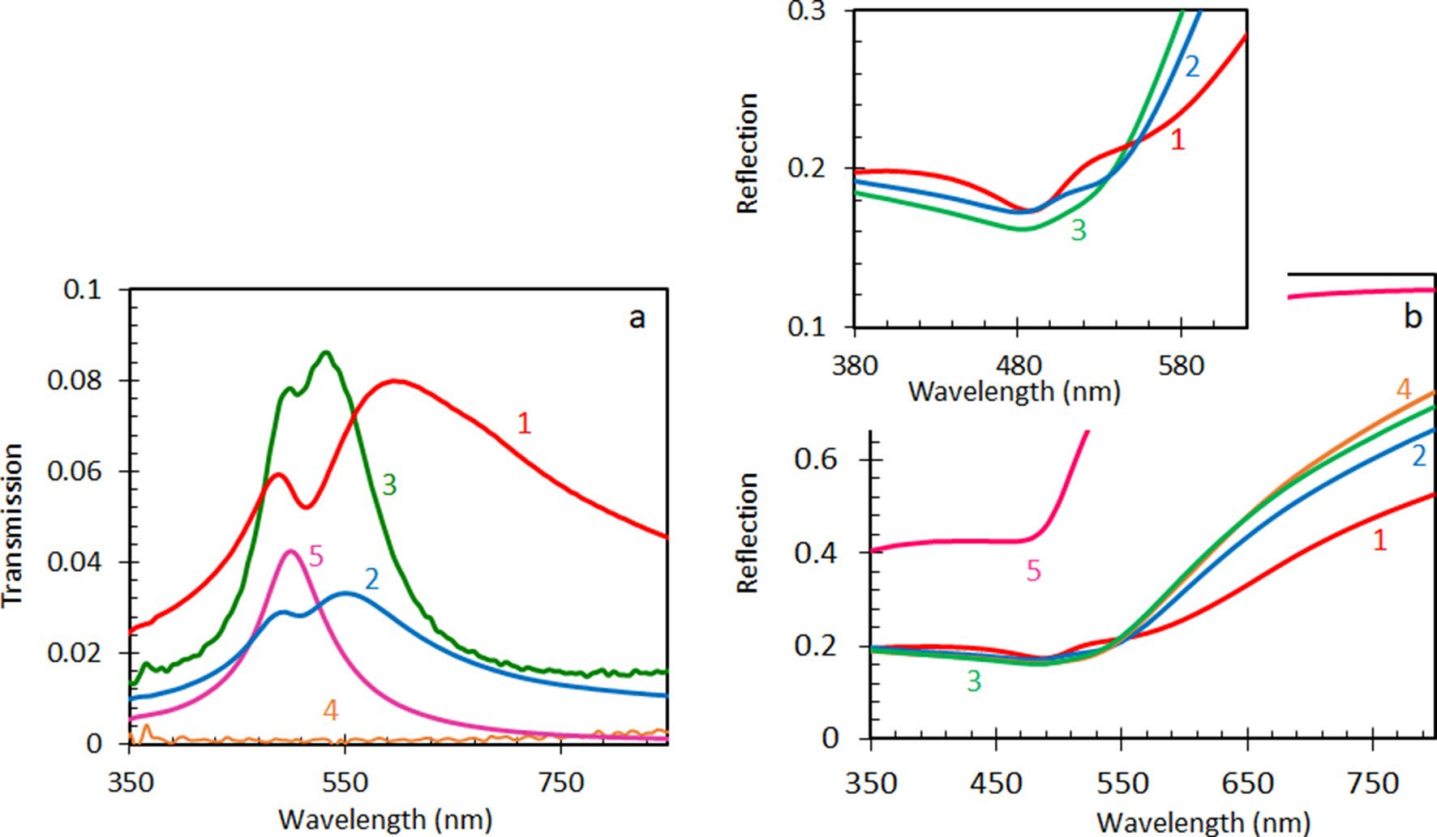
Transmission (**a**) and reflection (**b**) spectra of a single-layer (trace 1), two-layers (trace 2), four-layers (multiplied by 100, trace 3) and eight-layers (multiplied by 100, trace 4) NPGLs. Control sample: 90 nm Au film (trace 5). Inset: zoomed part of (**b**).

The results of the transmission and reflection measurements of the single-layer and multi-fold Au nanoleafs studied (taken using the Lambda 900 spectrophotometer from PerkinElmer) are summarized in Fig. 2a, b. The reference sample, 90 nm thick homogeneous gold film deposited on glass (using the thermal vapor deposition apparatus, Nano 36 from Kurt J Lesker), has the transmission maximum at 505 nm, where the real part of the dielectric permittivity of Au, *ε*′, approaches the epsilon-near-zero (ENZ) range^55,56^. At the same time, Au nanoleaf samples have two transmission peaks. The first one has its maximum almost at the same wavelength as the transmission peak in a thermally deposited smooth and homogeneous Au film, while the second one is shifted to longer wavelengths (up to 605 nm), Fig. 2a. The characteristic “wiggles” could be seen in the reflection spectra of the nanoleaf samples at the wavelengths corresponding to both transmission maxima, Fig. 2b.

Note that a qualitatively similar double-peak transmission spectra were obtained when Au films were deposited onto nanoporous anodic alumina membranes (pore diameter ~ 30 nm, Au film thickness 54 nm, membrane thickness ~ 50 µm, commercially acquired from Redox Inc.), Supplementary Information Fig. SI1. However, this phenomenon was not universal, and the transmission spectra of Au films deposited onto polymeric (PMMA) films with high concentration of microscopic air bubbles did not have two maxima in their transmission spectra (to be published elsewhere).

**Figure 3:**
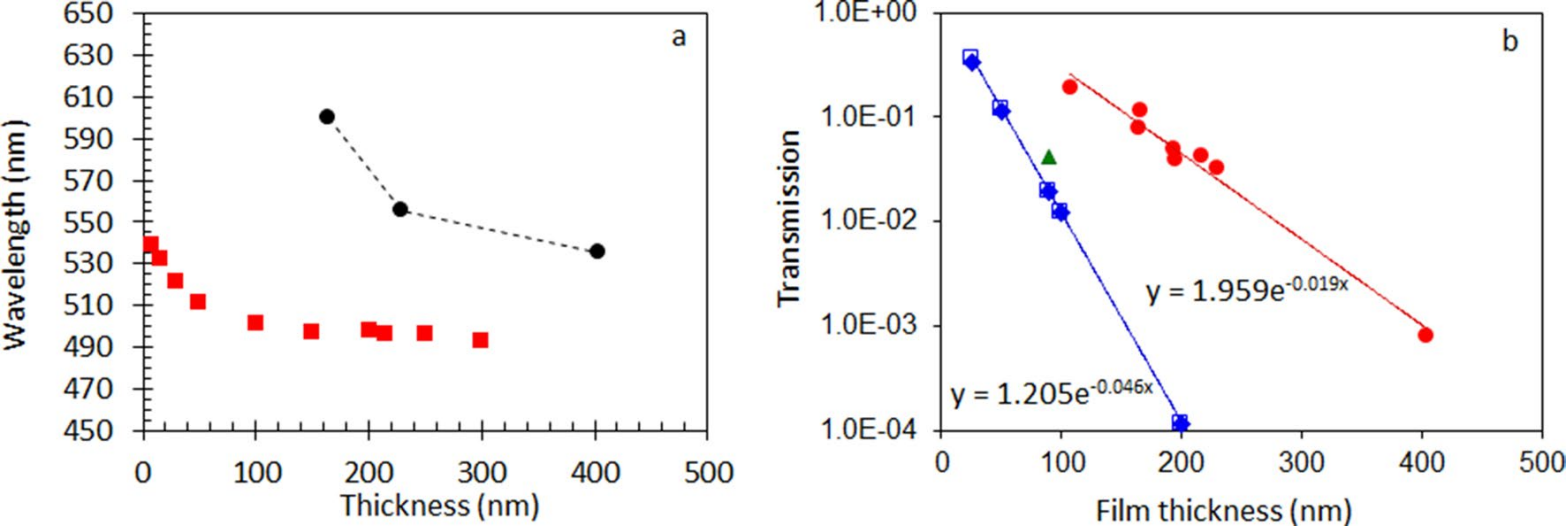
(**a**) Wavelength positions of the maxima of the transmission peaks; red squares—calculation done (using the online solver^57^) for homogeneous Au films; black circles—experimental measurements in porous Au nanoleafs. (**b**) Red circles: Transmission as the function of the thickness of the NPGL sample, experimentally measured in the maximum at *λ* ~ 590 nm. Open blue squares: Transmission as the function of the thickness of the homogeneous Au film (characterized by the dielectric permittivities)^55,56^, calculated^57^ at the spectral position of the long-wavelength transmission maximum. Solid blue diamonds: Transmission as the function of the thickness of the homogeneous Au film (characterized by the dielectric permittivities)^55,56^, calculated^57^ at *λ* = 498 nm. Green triangle: transmission measured in the maximum (at *λ* = 505 nm) in the homogeneous 90 nm Au film.

With increase of the number of layers and the overall thickness of the porous Au nanoleaf samples, the width of the long-wavelength transmission peak got reduced and its spectral position shifted to shorter wavelengths, Fig. 3a. The spectral shifts of the short-wavelength transmission peak and the dip between the two peaks were less significant. The transmission (measured in the maximum of the long-wavelength peak at *λ* ~ 590 nm) decreased with the increase of the sample’s thickness *d* almost exponentially, with the characteristic penetration depth of *l* = 53 nm, Fig. 3b.

**Figure 4:**
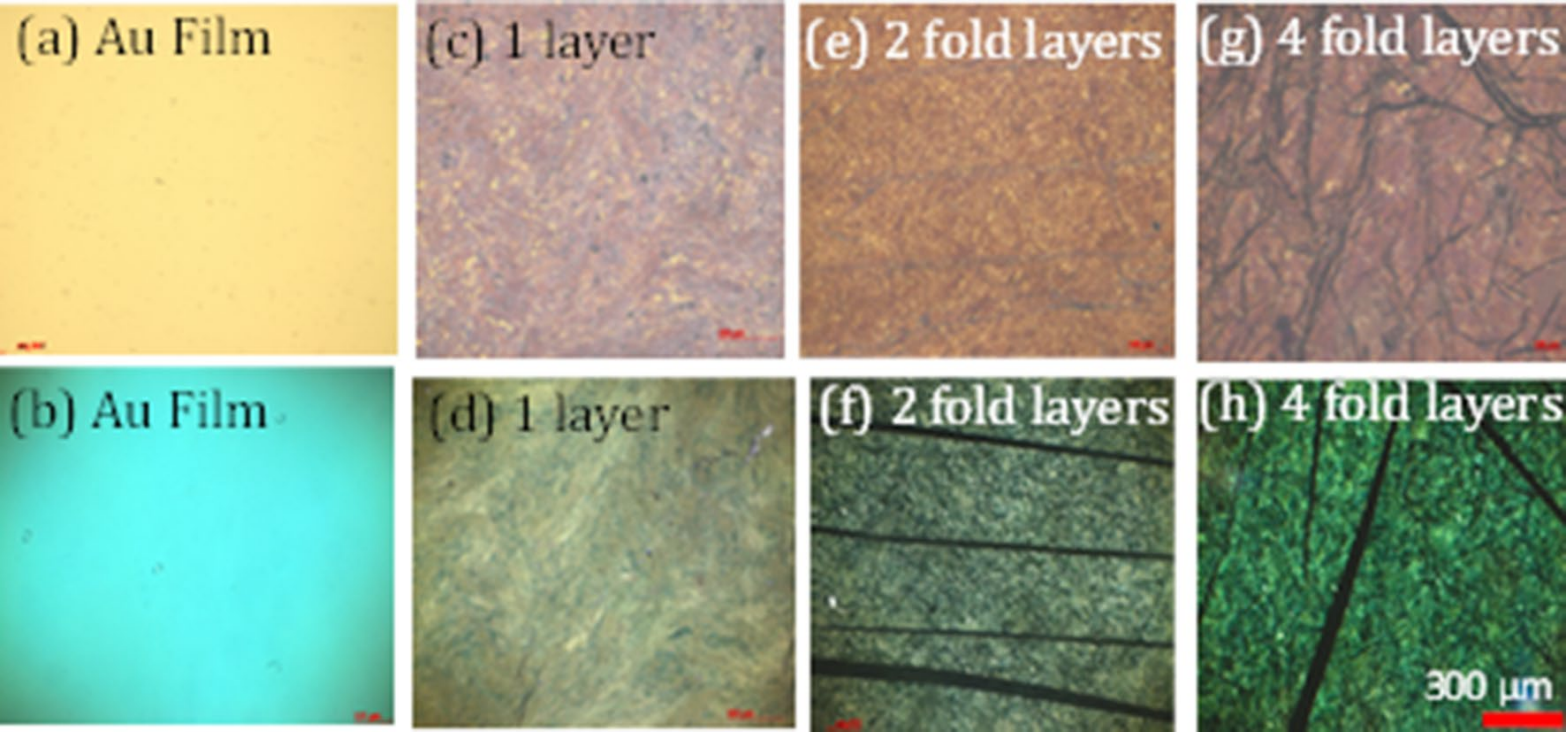
Microscopic images of the smooth 90 nm Au film (**a**,**b**), single-layer Au nanoleaf (**c**,**d**), two-fold Au nanoleaf (**e**,**f**), and four-fold Au nanoleaf (**g**,**h**) taken in the transmission (**b**,**d**,**f**,**h**) and reflection (**a**,**c**,**e**,**g**) modes of the optical microscope.

The transmission and reflection spectra depicted in Fig. 2a, b are in a good agreement with the color images of the same NPGL samples taken using the AxioVision 4 optical microscope (from Carl Zeiss) operating in the transmission and reflection regimes, Fig. 4. In fact, the light transmitted by the homogeneous thermally deposited Au film and four-fold Au nanoleaf (featuring short-wavelength transmission peaks) has a greenish-bluish color, while the light transmitted by the single-layer Au nanoleaf (characterized by a relatively long-wavelength transmission) is more yellowish. Dark lines, which are particularly pronounced in Fig. 4f–h are wrinkles in multi-fold samples.

### Effect of the dielectric environment

**Figure 5:**
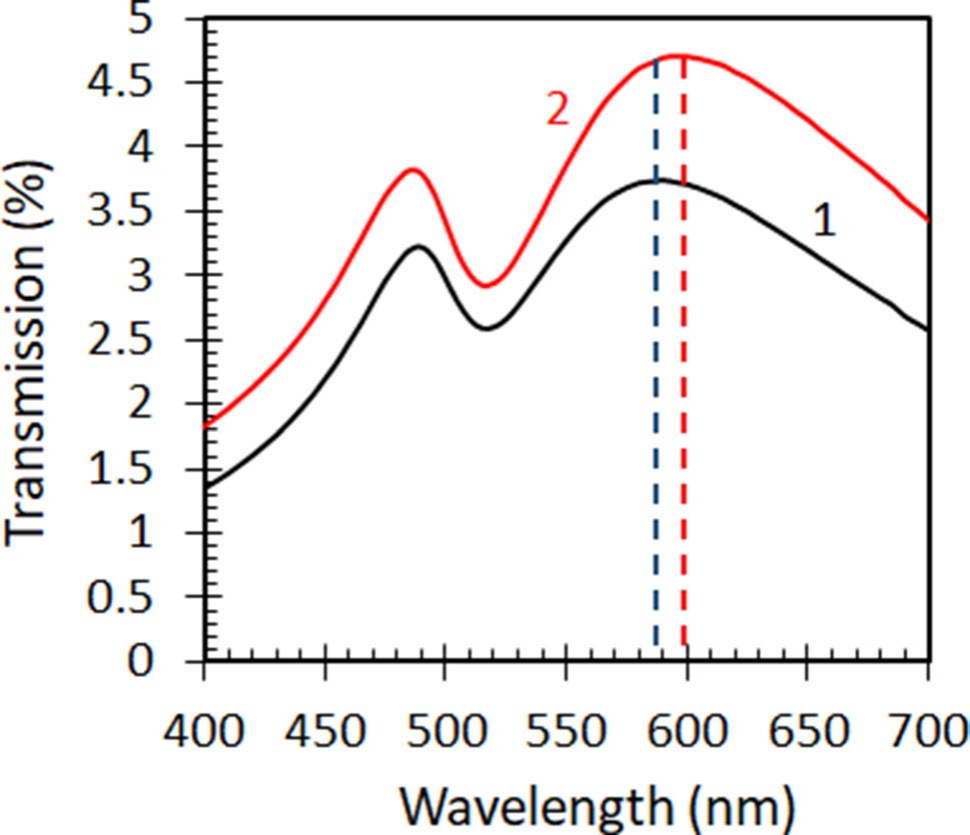
Transmission spectra of the Au nanoleaf in air (trace 1) and in water (trace 2).

Our next particular experiment was aimed at control of the optical properties of NPGLs with external dielectric environments. When Au nanoleaf was placed in water, its long-wavelength absorption peak moved slightly (by ~ 10 nm) to shorter wavelengths, while the short-wavelength peak and the dip between the two peaks practically did not change their spectral positions, Fig. 5. This result is in a qualitative agreement with the literature^50^. However, the spectral shift in our studies is smaller than that reported by Lang et. al.^50^ On the other hand, the two transmission peaks in Ref.^50^ could be observed only when the NPGL samples were immersed in a liquid, and only one transmission peak was seen in a dry sample, without any liquid. The effect of water on the transmission of NPGLs can be explained by change in the effective medium’s dielectric permittivity and/or spectral shift of surface plasmon resonances (supported by Au ligaments) in the presence of water.

### Control of transmission with applied voltage

**Figure 6:**
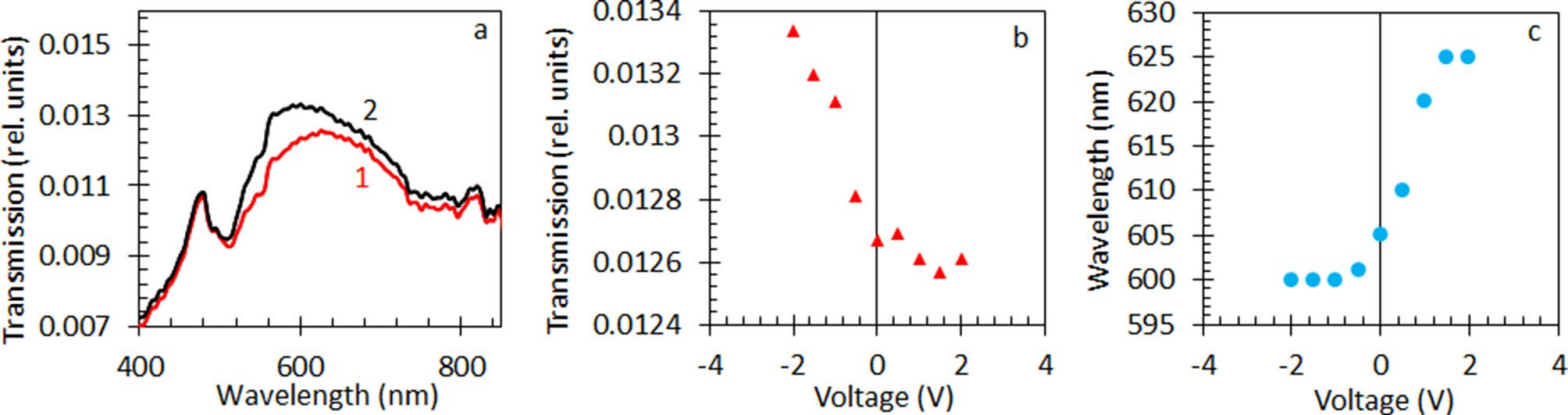
(**a**) Transmission spectra of Au nanoleaf in a two-electrode electrochemical cell at positive voltage (+ 1.5 V, trace 1) and negative voltage (− 2 V, trace 2) applied to the NPGL working electrode. (**b**) Transmission peak intensity as a function of the applied voltage. (**c**) The wavelength of the transmission peak as a function of the applied voltage.

To tune the optical properties of NPGLs in real time, we placed the sample in the two-electrode electrochemical cell. The NPGL sample, with attached copper tape conduit, served as a working electrode, while a platinum wire played the role of a counter electrode. Following Ref.^49^, the electrolyte was 0.7 M solution of NaF in water. The applied voltage varied between − 2 V and + 2 V (applied in a sequence 0 V, + 0.5 V, − 0.5 V, + 1 V, − 1 V, …), caused changes in both the strength (~ 10%) and the wavelength position (Δλ ~ 20 nm) of the long-wavelength transmission peak, Fig. 6a–c. No permanent damage occurred to the sample until + 2.5 V, after which (at − 2.5 V) the Au nanoleaf got damaged and detached from the glass substrate. The voltage-induced changes in the optical spectra were, presumably, due to change in the carriers’ density^49^, which, in turn, affected the effective medium’s dielectric permittivity and plasmonic resonances of Au ligaments. Note that the largest spectral changes in our experiment were observed when negative (rather than positive)^49^ voltage was applied to the gold leaf. This is not the same as (although not in direct contradiction with) the result of Ref.^49^, where the strongest changes occurred in the transmission spectra, when the applied potential (measured relative to the Ag/AgCl pseudo-reference electrode) was positive. This intriguing phenomenon requires more studies, which results will be published elsewhere.

### Familiar and unfamiliar gold

**Figure 7:**
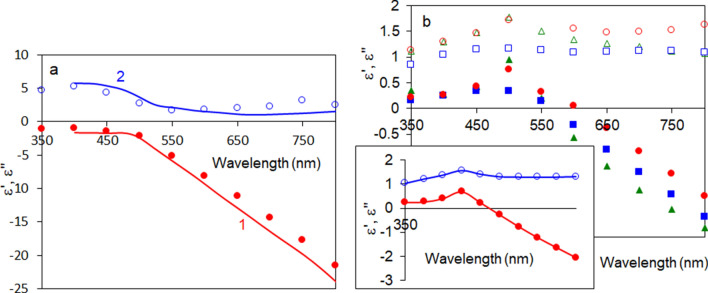
(**a**) Spectra of the real (red trace 1 and solid circles) and imaginary (blue trace 2 and open circles) parts of the dielectric permittivity of Au. Solid line—the data from Refs.^55,56^, characters—the data derived from the experiment. (**b**) Spectra of real (closed characters) and imaginary (open characters) parts of dielectric permittivity of a single-layer Au nanoleaf (164 nm, red circles), two-fold Au nanoleaf (230 nm, blue squares) and four-fold Au nanoleaf (404 nm, green triangles). Inset: average of the three spectra of dielectric permittivities depicted in the main frame; red closed circles—real parts of dielectric permittivity *ε*′, blue open circles—imaginary parts of dielectric permittivity *ε*″.

**Figure 8:**
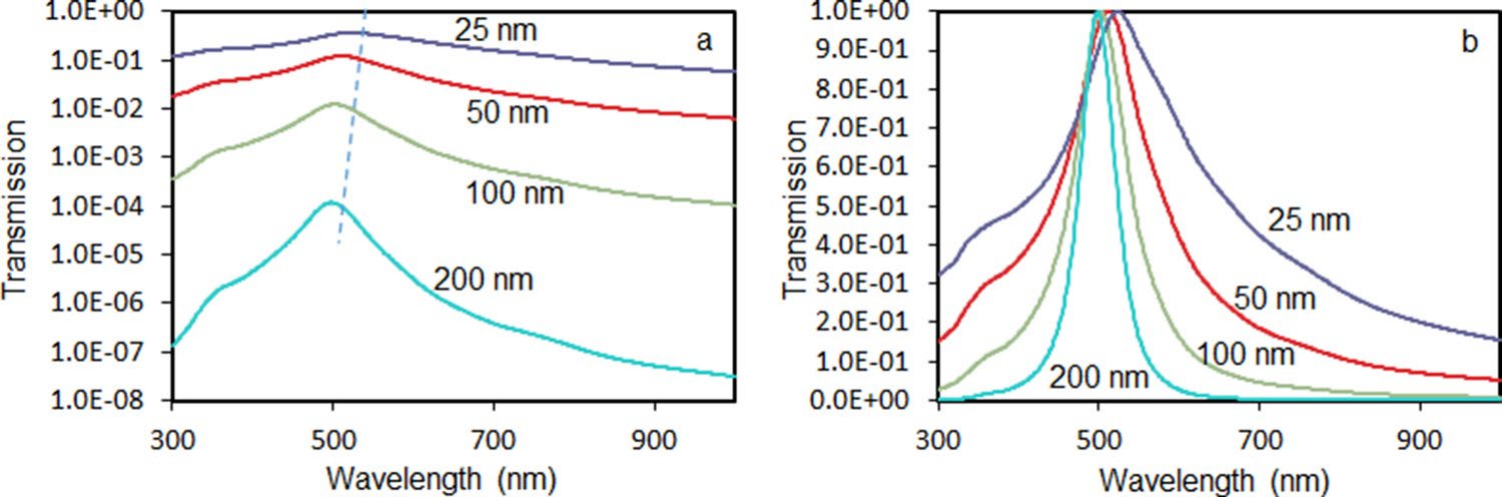
Normal-incidence transmission spectra of Au films of different thickness, computed using the online solver^57^, for the dielectric permittivities of gold reported in Refs.^55,56^, (**a**) semi-logarithmic vertical scale, dashed line indicates the shift of the transmission maxima; (**b**)—linear vertical scale, emission maxima are normalized to unity.

Before trying to understand the rather unusual double-headed transmission spectra of NPGLs, let us review a seemingly familiar transmission spectrum of a smooth and homogeneous Au film deposited on glass. We assumed gold to have the dielectric permittivities similar to those of Refs.^55,56^ (Fig. 7a) and calculated, using the online solver^57^, the series of normal-incidence transmission spectra for Au films, whose thicknesses ranged between 25 and 200 nm, Fig. 8a,b. As one can see, the transmission spectra of homogeneous gold films are characterized by a single peak, whose magnitude (Figs. 3b and 8a) and width (Fig. 8b) are getting smaller and the spectral position shifts to shorter wavelengths (Figs. 3a and 8a) with increase of the film’s thickness. Therefore, the only important difference between the transmission spectra of homogeneous thermally deposited Au films and porous Au nanoleafs is the number of peaks. Otherwise, the behavior of the λ ~ 590 nm transmission peak observed in NPGL is qualitatively similar to that of the transmission peak in a homogeneous Au film.

In the homogeneous gold film, the thickness dependence of the maximal transmission intensity (and nearly similar to it transmission measured at *λ* = 498 nm) is nearly exponential, with the penetration depth equal to 22 nm, Fig. 3b. (The green triangle in Fig. 3b is the experimental point.) Besides the number of the transmission peaks (one or two), this behavior is in a qualitative agreement with that of NPGLs, Fig. 3b. The fact that the sample’s extinction is fairly described by an exponential Beer’s law and, at the same time, the extinction spectral maximum changes with an increase of the film’s thickness, is counterintuitive. It can be explained by the fact that the extinction, which is measured in the experiment, is determined not only by the sample’s absorption, but also by the strong reflection, which does not follow the Beer’s law.

### Dielectric permittivities of porous Au nanoleafs

The reflection *R* (*λ*) and transmission *T*(*λ*) spectra of three-layered sandwich-like structures can be calculated using the analytical formulas derived in Ref.^5^ and knowing the complex dielectric permittivities (*ε*′ + i *ε*″) of all three constituent media: *ε*′, *ε*″ → *R*, *T*. Solving (numerically) the inverse problem, one can derive the spectra of real and imaginary parts of dielectric permittivities, *ε*′ (*λ*) and *ε*″ (*λ*), if *R*(*λ*) and *T*(*λ*) are known: *R*, *T* → *ε*′, *ε*″. Note that this point-by-point calculation does not depend on any knowledge or assumptions regarding the relationships between the dielectric permittivities at different wavelengths and/or Drude *versus* Lorentz nature of the spectra. When we applied this technique to the 90 nm homogeneous Au film deposited on glass, we obtained a good agreement between the derived dielectric permittivities and those of Refs.^55,56^, compare characters and solid lines in Fig. 7a. The minor mismatch between the latter two datasets can have many possible reasons; one of them is that our Au films were not exactly the same as the gold surfaces studied in reference^55^.

We further applied the same extraction method to a single-layer (164 nm), two-fold (230 nm), and four-fold (404 nm) porous Au nanoleafs and obtained the spectra of dielectric permittivities, which could be described as the spectra of “diluted” gold, compare Fig. 7a,b. Expectedly, the spectra of *ε*′ and *ε*″ practically did not depend on the number of layers or the overall thickness of the NPGL samples. The spectra of the real and imaginary parts of dielectric permittivity, averaged over three samples studied, are depicted in the inset of Fig. 7b. Note that the derived spectra of *ε*′ and *ε*″ are in a good agreement with those of uncoated and not annealed thick (~ 200 µm) Au nanofoam samples, fabricated using a similar de-alloying technique^47^.

### The toy model of NPGL

As we have discussed above, many optical properties of NPGLs are qualitatively similar to those of homogeneous Au films. Thus, with increase of the film’s thickness, the transmission peak narrows and shifts to shorter wavelengths, its intensity decreases nearly exponentially, and the spectra of dielectric permittivities of NPGLs resemble those of “diluted” gold. The only vast difference between the transmission spectra of homogeneous and porous Au films is that the former has one peak while the latter has two. Although surface plasmons have been named in the literature as a plausible reason for the double-peaked transmission spectra of NPGLs^49,50,52^, the underlining physics of this intriguing phenomenon has never been clearly explained.

**Figure 9:**
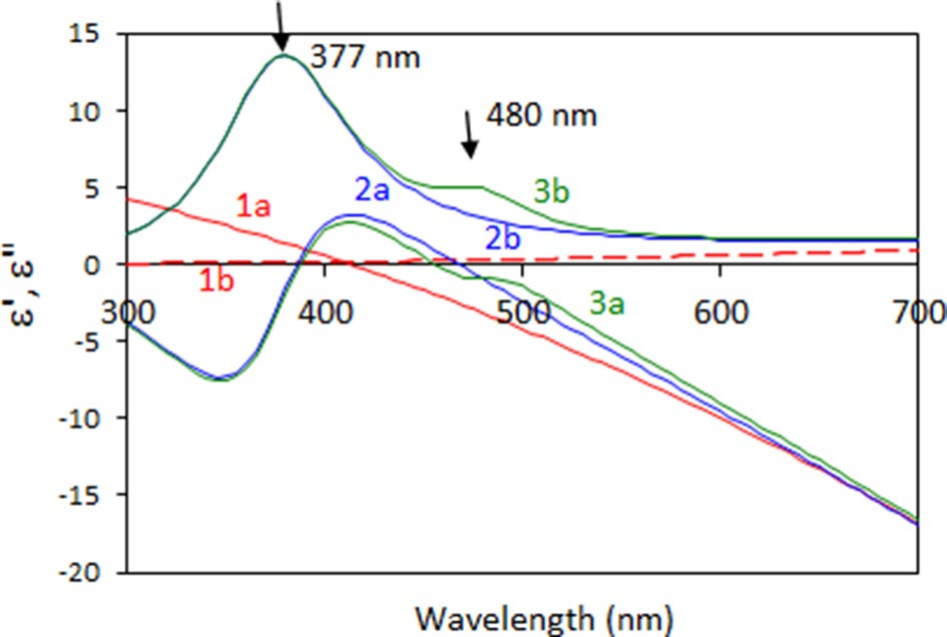
Spectra of dielectric permittivities used in the toy model; 1a and 1b—Drude terms (Eq. 1); 2a and 2b Drude and single Lorenzian; 3a and 3b—Drude and two Lorentzians. 1a, 2a, and 3a—real parts of dielectric permittivities; 1b, 2b, and 3b—imaginary parts of dielectric permittivities.

**Figure 10:**
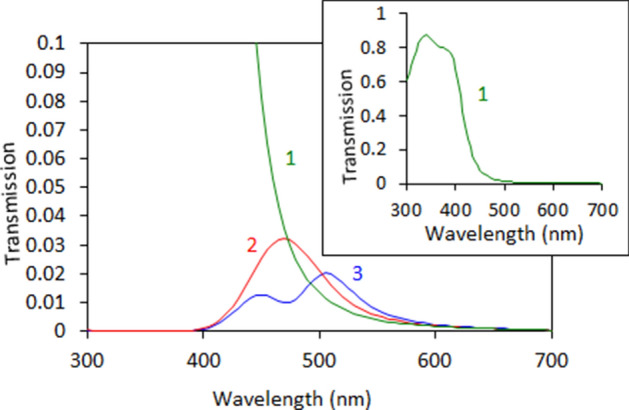
Transmission spectra calculated using the dielectric permittivities determined by the Drude model (trace 1), Drude + single Lorenzian model (trace 2), and Drude + two Lorenzians model (trace 3).

Below, we introduce a simple toy model qualitatively explaining the double-headed transmission spectra of porous Au nanoleafs. We start with the Drude model for Au (first two terms on the right-hand side of Eq. 1),

1 $$ \varepsilon = \varepsilon_{b} + \frac{{\omega_{Dp}^{2} }}{{ - \omega^{2} - i\omega \gamma_{D} }} + \frac{{L_{1} }}{{\omega_{{L_{1} 0}}^{2} - \omega^{2} - i\omega \gamma_{{L_{1} }} }} + \frac{{L_{2} }}{{\omega_{{L_{2} 0}}^{2} - \omega^{2} - i\omega \gamma_{{L_{2} }} }} $$

where *ε*_*b*_ = 9 is the bulk dielectric permittivity of Au, ω is the angular frequency, *ω*_Dp_ = 1.37 × 10^16^ rad/s is the Drude plasma frequency (determined by the concentration of free electrons), and *γ*_*D*_ = 1 × 10^14^ rad/s is the free electrons' dissipation rate^58^. The corresponding spectra of real and imaginary parts of dielectric permittivity are shown in Fig. 9 traces 1a and 1b). The real part of the dielectric permittivity *ε*′ crosses zero at *λ* = 415 nm, causing a very large reflection and, correspondingly, very small transmission at longer wavelength, Fig. 10 (trace 1). At the same time, both absorption and reflection are small at shorter wavelengths, resulting in a very broad and strong transmission band, which does not resemble the transmission spectra of neither homogeneous nor nanoporous Au films.

The latter shortcoming of the Drude model (caused by neglecting bound state electron transitions, which are particularly strong in the blue-to-ultraviolet range of the spectrum) is well known in the literature. In order to overcome it, we added to the model the Lorenzian band (third term in the right-hand side of Eq. 1) simulating the bound state transitions, with the maximum at *λ* = 377 nm, Fig. 9 (traces 2a and 2b). (Here $\omega_{{L_{1} 0}}^{2}$ is the natural frequency of oscillation, $\gamma_{{L_{1} }}$ is the dissipation rate, and *L*_1_ is the amplitude factor determined by the concentration of the oscillating electrons and the oscillator strengths.) Now the strong Lorentzian absorption band restricts the transmission at short wavelengths, while the strong reflection at *λ* ≥ 415 nm still limits it at long wavelengths. The narrow spectral window determined by these two boundaries constitutes the (single-headed) transmission peak observed in homogeneous Au films, Fig. 10 (trace 2).

In order to explain the origin of the two maxima in the transmission spectra of porous Au nanoleafs, let us recall that gold nanospheres and nanorods support localized surface plasmon resonances in vicinity of 500 nm^49^, and gold ligaments composing NPGLs should not be an exception. Therefore, in accord with Ref.^49^, a surface plasmon absorption band is expected in the spectral range of the gold’s transmission window. If the latter absorption band is strong enough and narrow enough, it can create a dip in the middle of a broader transmission spectrum, causing the double-headed transmission band observed it in the spectra of Au nanoleafs.

In order to illustrate this, we added to Eq. (1) the second Lorenzian band with the maximum at 480 nm, the fourth term in the right-hand side (the notations are the same as above). This allowed us to model the spectrum of dielectric permittivities Fig. 9 (traces 3a and 3b), which resulted in the dip in the middle of the broader transmission band, Fig. 10 (trace 3). This shape, a broad band with a dip in the middle, can be alternatively described as a double-headed transmission peak. (Note that the width of the second Lorenzian band in the model was commeasurable with that determined experimentally.^52^) Although the Lorenzian shape of the latter surface plasmon absorption band is not well justified, the maximum at *λ* ~ 500 nm can be seen in the experimental spectra of dielectric permittivities of NPGLs, see inset of Fig. 7b. (We infer that almost any bell-shape absorption band can be used in the calculations for illustration purposes.) Although the toy model above is not rigorous or comprehensive, it qualitatively interprets the highly unusual double-headed transmission band of NPGLs as one broad transmission peak with the dip in the middle.

## Summary

To summarize, we have studied optical properties of single and multi-fold nanoporous gold leafs (NPGLs) of different thickness and found that with increase of the leaf’s thickness, the transmission spectrum narrows and shifts to shorter wavelengths and its intensity decreases nearly exponentially—the behavior, which is qualitatively similar to that of homogeneous gold films. Furthermore, the dispersion of dielectric permittivities of NPGLs resembles that of “diluted” gold. The vast difference between the transmission spectra of homogeneous and porous Au films is that the former has one maximum while the latter has two. We explain this intriguing phenomenon in terms of a relatively narrow surface plasmon absorption band positioned on top of the broader transmission band. We further demonstrated that the optical properties of NPGLs can be controlled by applied voltage in an electrochemical cell (presumably due to change in the electron density^49^) and by change of the dielectric environment, e.g., by immersing Au nanoleafs in water^50^. Although we were able to qualitatively explain the major experimental results, the underlying fundamental physics and applications of the transmission, reflection, dispersion, and emission (not discussed here) of NPGLs require further studies to be published elsewhere.

## Supplementary Information

Supplementary Information 1.
