# Supplementary material for: Nanoporous gold nanoleaf as tunable metamaterial
Source: Sci Rep. 2021 Jan 19;11:1795. doi: 10.1038/s41598-021-81128-4 (PMC7815877; doi:10.1038/s41598-021-81128-4)
Supplement: Supplementary file 1 — Supplementary Information 1. [file 41598_2021_81128_MOESM1_ESM.docx]

**Supplementary Information**

**Nanoporous Gold Nanoleaf as Tunable Metamaterial**

Sangeeta Rout^1^, Zhen Qi^2^, Monika M. Biener^2^, Devon Courtwright^1^, Jakeem C. Adrien^1^, Ezekiel Mills^3^, Mohammad Shahabuddin^1^, Natalia Noginova^1^ & Mikhail A. Noginov^1*^

^1^Center for Materials Research, Norfolk State University, Norfolk, VA 23504, USA

^2^Lawrence Livermore National Laboratory, Livermore, CA 94550, USA

^3^ *Virginia State University, Petersburg, VA 23806, USA*


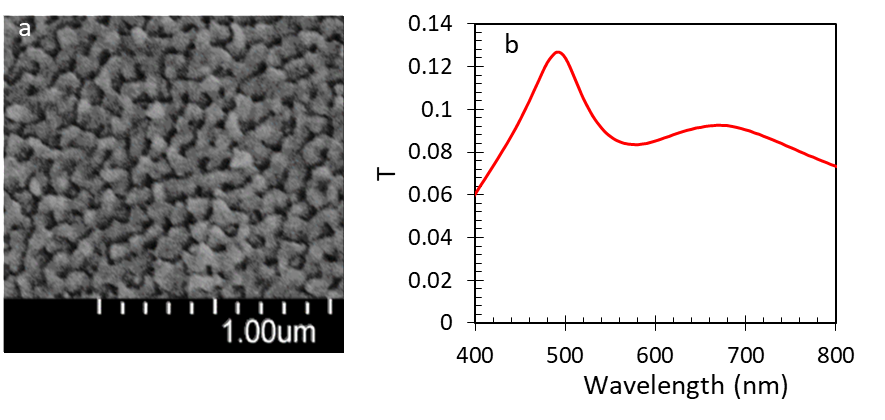


**Figure SI1.** (**a**) Typical SEM image of an anodic alumina membrane coated with a gold film. (**b**) Transmission spectrum of the gold coated alumina membrane (film thickness 54 nm, pore diameter 30 nm).
